# Cognitive Deficits in Alcoholic Women

**Published:** 1994

**Authors:** Sara Jo Nixon

**Affiliations:** Sara Jo Nixon, Ph.D., is the director of the Cognitive Studies Laboratory, Oklahoma Center for Alcohol and Drug Related Studies, and an associate professor in the Department of Psychiatry and Behavioral Sciences, University of Oklahoma Health Sciences Center, Oklahoma City, Oklahoma

## Abstract

Long-term alcohol abuse can impair the brain’s intellectual and problem-solving functions. Research suggests that women may be more sensitive than men to this impairment.

Research indicates that long-term alcohol abuse^1^ can impair virtually all of the brain’s information-processing (cognitive) functions. Much of this research has been limited to the study of alcoholic men, perhaps, in part, because for many years, the primary locations for such research were Veterans’ Administration hospitals. This situation has changed in recent years. With increasing treatment availability and changing social mores regarding alcoholism, more women are obtaining treatment, thereby becoming available for study.

This article focuses on the neuropsychologic and neurophysiologic consequences of long-term alcohol abuse in women. Broadly defined, neuropsychologic functions include learning, memory, abstract thinking, problem-solving, perceptual-motor skills (such as eye-hand coordination), and the ability to analyze spatial relationships (Hunt and Nixon 1993). Neurophysiologic functions are related to the structure and electrical activity of the brain.

## Neuropsychologic Deficits in Alcoholic Women

A pioneering assessment of alcoholic women and men was conducted by Acker (1986), who used a battery of paper-and-pencil and computer tests to assess problem-solving ability involving spatial relationships. The performance of alcoholics was matched to that of nonalcoholic control subjects of similar educational level, age, and intelligence. Alcoholics of both genders performed poorly compared with control subjects. However, alcoholic women performed as poorly as alcoholic men, even though the latter reported more years of alcoholic drinking.

Bergman (1987) used a battery of standard tests to assess neuropsychologic performance in male and female alcoholics. Control subjects were selected randomly from a national register of all inhabitants of Sweden. Tests included the Halstead-Reitan Neuropsychological Test Battery, which provides an index of overall neuropsychologic impairment. The higher the index (range 0.0 to 1.0), the greater the impairment. These findings revealed modest (0.5 to 0.8) to profound (0.9 to 1.0) impairment in 22 percent more of the alcoholic than the control men and 24 percent more of the alcoholic than the control women. Alcoholic women reported approximately one-half the duration (years) of heavy drinking and consumed about 37 percent less alcohol per drinking occasion as alcoholic men. These findings are consistent with Acker’s conclusion (1986) that women are more sensitive than men to the cognitive effects of long-term alcohol abuse.

Since these early studies, considerable research has been performed on the nature of alcohol-related cognitive deficits in women. Most results suggest that male and female alcoholics share a common pattern of neuropsychologic dysfunction despite alcoholic women typically reporting shorter or less severe alcoholic drinking patterns than alcoholic men (Parsons 1993; National Institute on Alcohol Abuse and Alcoholism 1990).

**Figure 1:**
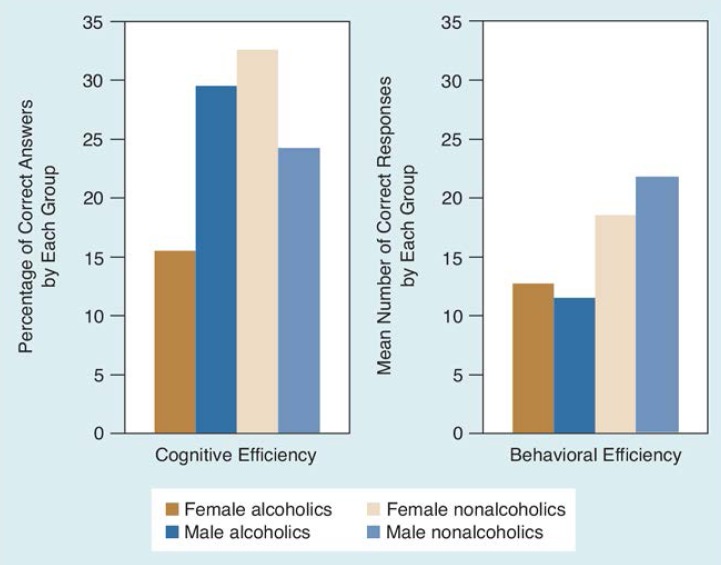
Performance of alcoholic and nonalcoholic men and women in tests of four types of neuropsychologic functioning. The bars represent the mean performance of each group of subjects for each type of functioning. For comparison purposes, performance results were standardized at a mean score of 50. ^1^Set-shifting flexibility is typified by the ability to change problem-solving strategies in response to changing requirements.

This conclusion is supported by a recent study of men and women performing a wide range of neuropsychologic tests (Glenn et al. 1993). Four domains were assessed: verbal skills, such as vocabulary and word finding; visual-spatial performance, such as reconstruction of block designs; verbal memory, such as memory for stories; and set-shifting flexibility, such as the ability to change problem-solving strategies in response to changing requirements. Groups of alcoholics and controls, equivalent to one another in terms of age and education, then were compared with respect to these factors. Across all domains, alcoholics, regardless of gender, performed more poorly than controls (figure 1).

The studies discussed above demonstrate the pervasive nature of the cognitive effects of long-term alcohol abuse. On the individual level, however, an alcoholic may perform poorly on one task while doing well on others. Moreover, alcohol does not always affect all neuropsychologic functions equally. Whereas alcoholics frequently demonstrate poorer abstraction and problem-solving skills compared with controls, deficits in verbal learning and memory are observed less consistently (see Parsons 1993).

Several hypotheses have been put forth to account for these observations; however, a comprehensive discussion is beyond the scope of this article (see Oscar-Berman 1987). In summary, it appears that the mild generalized brain dysfunction hypothesis best accounts for the existing data for both alcoholic men and alcoholic women. This hypothesis states that long-term alcohol abuse produces a mild to moderate, non-specific, highly variable pattern of overall neuropsychologic impairment (Oscar-Berman 1987). A limitation of this hypothesis, according to some researchers, is that it fails to suggest directions for further research.^2^ Nevertheless, it accurately describes the experimental observations regarding alcoholic neuropsychologic performance (Nixon 1993).

## Deficits That May Underlie Poor Cognitive Performance

Instead of measuring performance on specific tasks, some investigators have examined the underlying generalized cognitive processes that may be affected by long-term alcohol abuse (e.g., Smith and Oscar-Berman 1992). Our laboratory has focused on investigating cognitive efficiency, defined here as the ability to attend to relevant accurate information while ignoring irrelevant or inaccurate information (Nixon 1993).

In one set of these experiments, we examined the speed and accuracy of the subjects’ performance on an extended form^3^ of the battery of tests used by Acker (1986), as discussed earlier. We found that groups of sober alcoholic men and women took longer than groups of nonalcoholic control subjects, who were equivalent in age and education to the alcoholics, to complete the tests. However, only alcoholic women were less accurate than their matched controls. When efficiency ratios (accuracy divided by time) were considered, alcoholic men and women were equally impaired (Glenn and Parsons 1990). A later study using this same approach also found significant alcohol-related deficits in efficiency measures (Glenn and Parsons 1992). As in the previous study, alcoholic men and women performed similarly despite shorter durations of alcoholism among the women.

It has been argued that laboratory tests are so sensitive to subtle impairment that performance on these tests might not reflect performance in real-world situations. To address this concern, we administered the “plant task” (reviewed in Nixon 1993), which requires subjects to identify and isolate the relevant aspects of a problem that might be met in real life.

Subjects are shown four plants; two of the plants are healthy, two are sickly. Each plant is associated with a different combination of treatment factors, including different amounts of water (large vs. small glass), presence or absence of leaf lotion, and type of plant food (blue or yellow). The experimenter describes the treatment each plant has received. The plants remain in full view throughout the test, each surrounded by the bottles and containers that represent its particular treatment combination. The experimenter then displays the treatment combination intended for a fifth, unseen plant. Subjects are asked first how they believe the unseen plant will fare under that treatment and second how they came to that conclusion.

The correct answer to the first question is “well” or “healthy.” Moreover, only one treatment factor, the type of plant food, predicts plant health. Thus, the correct answer to the second question is “because it receives the blue plant food” (or something similar).

Analysis of responses indicated that alcoholics, regardless of gender, were able to identify the correct factor but failed to isolate it from other factors. That is, alcoholics’ responses often combined the correct answer with irrelevant factors, such as, “Well, the plant food has something to do with it, but I’d give it a little more water as well.” This inability to ignore the irrelevant constitutes a cognitive inefficiency.

## Interpersonal Relationships

Despite the theoretical and practical importance of the studies described above, they fail to address a critical area of alcohol-related cognitive impairment: interpersonal relationships. This area can be addressed using the Adaptive Skills Battery (Jones and Lanyon 1981), a series of 30 short situations to which respondents must provide either what they believe would be their “typical” response or what they believe is the “best” response to the situation. Individual items consider such issues as peer pressure to use alcohol, children who violate curfew, and difficult work and home situations. Scores to responses on any one item may range from 0 (e.g., use of force or drugs as a solution to the problem) to 3 (e.g., use of negotiation, appropriate distancing from the problem).

Alcoholic men have been shown to have lower scores on the “typical” responses than do nonalcoholic men (Patterson et al. 1988). However, the groups did not differ on their “best” responses. In 1992 we administered this test to alcoholics (18 men and 16 women) and nonalcoholics (15 men and 12 women) (Nixon et al. 1992). Data supported the earlier finding: alcoholics had lower scores on the “typical” responses than nonalcoholics but did not differ from nonalcoholics in their “best” responses. The differences were not attributable to differences in age, education, or gender. Thus, it appears that alcoholics are aware of the more appropriate response in situations of interpersonal conflict but fail to enact those responses. We have referred tentatively to this pattern of cognitive awareness coupled with behavioral inattention as an indicator of behavioral inefficiency. Additional research is needed to replicate and clarify this pattern.

**Figure 2:**
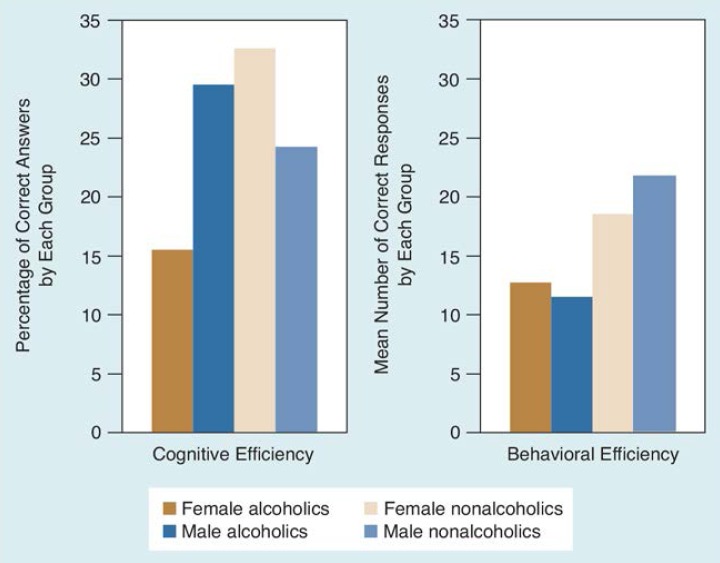
Measures of cognitive^1^ and behavioral^2^ efficiency in alcoholic and non-alcoholic men and women. The graph at the left illustrates results from a test of cognitive efficiency (the “plant test”). The numbers refer to the percentage of correct answers for each group. Although female alcoholics performed more poorly than other subjects, the only statistical difference was between alcoholics as a group and nonalcoholics. The graph at the right illustrates a component of behavioral efficiency. Numbers are average ratings for each group. Alcoholics’ responses were significantly poorer than nonalcoholics’ responses. ^1^Cognitive efficiency is the ability to attend to relevant, accurate information while ignoring irrelevant or inaccurate information. ^2^Behavioral efficiency refers to the enactment of appropriate responses in situations of interpersonal conflict.

Figure 2 summarizes the efficiency deficits for these two studies, illustrating that alcoholic men and women demonstrate significant impairment on ecologically valid (“real-world”) tasks, consistent with results of more conventional laboratory and clinical research. A broad definition of efficiency incorporating the concept of ignoring irrelevancy may therefore be a useful way of assessing alcohol-related deficits.

## Neurophysiologic Changes in Alcoholic Women

Long-term alcohol abuse is associated with alterations in the electrical and structural properties of the brain (for reviews, see Hunt and Nixon 1993). Relevant research has been limited to male subjects to an even greater extent than has the research on neuropsychologic functioning. Studies that included women have produced inconsistent results, as discussed below.

### Event-Related Potentials

Event-related potentials (ERP’s) are electrical “brain waves” that occur in response to external stimuli. The timing and intensity of brain waves are measured by the electroencephalogram. The P300 is an ERP component occurring approximately 300-thousandths of a second after stimulus onset. Parsons and colleagues (1990) studied ERP’s obtained in response to auditory and visual stimuli in alcoholic and nonalcoholic men and women. Consistent with other data (Porjesz and Begleiter 1993), alcoholic men demonstrated smaller (less intense) P300’s than did controls. However, alcoholic women did not differ from control women in this respect.

Hill and Steinhauer (1993) examined visual and auditory ERP’s in alcoholic women and their nonalcoholic sisters. Control subjects were nonalcoholic women recruited from the community. In contrast to the study by Parsons and coworkers, these data showed a significant association between alcoholism and reduced P300’s; that is, alcoholic women had significantly smaller P300’s than did either their sisters or the community controls. Based on this and other data, the researchers concluded that low P300 intensity may serve as a marker for assessing the risk for developing alcoholism in young women.

### Brain Shrinkage

Computed tomography (CT) is the first technology developed for producing three-dimensional images of structures within the body. Other three-dimensional imaging techniques have since been developed, including magnetic resonance imaging (MRI). CT employs X rays to produce images, whereas MRI uses magnetic fields. Both techniques are used commonly by clinicians as well as researchers to view the living brain (Zakhari and Witt 1992).

Jacobson (1986*a*) examined alcoholic women using CT and found enlarged ventricles (fluid-filled cavities within the brain) and widened sulci (furrows on the surface of the brain). Because the brain occupies a closed space within the skull, these findings indicate shrinkage of brain tissue. The degree of brain shrinkage detected in women was similar to that reported in the literature for alcoholic men, despite the women having reported lower peak alcohol consumption (based on self-reported highest daily consumption during a typical heavy drinking bout).

Similarly, a CT study by Mann and colleagues (1992) found equivalent levels of brain shrinkage in alcoholic men and women despite shorter drinking histories in alcoholic women.

In contrast to the above results, an MRI study by Kroft and colleagues (1991) found enlarged ventricles in only 1 of the 10 women studied (see also Pfefferbaum et al. 1992). This difference in results may be attributable to several factors, including the small sample size in the Kroft study, and differences in sensitivity between CT and MRI.

The gender-related differences might be reduced if alcohol amounts were equated for body mass/body water ratios. However, studies of chronic use typically have not obtained such information. Even if this adjustment could be applied, differences in the reported years of alcohol abuse would remain.

## Recovery of Cognitive Function

Neuropsychologic function tends to recover progressively in alcoholic men and women over approximately a 2- to 5-year period after abstinence (reviewed in Parsons 1993). Verbal skills appear to recover earliest, but subtle deficits, particularly in abstraction and problem-solving, may persist.

Continued abstinence is important for sustained recovery of neuropsychologic function. Alcoholic men and women who resume drinking, even at greatly reduced rates, remain cognitively impaired when retested. Some research suggests that those subjects who relapsed to drinking demonstrated poorer neuropsychologic performance initially than did those who remained abstinent; the significance of this finding is unclear (Parsons 1993).

Few studies of neurophysiologic recovery have been conducted with alcoholic women. Jacobson (1986*b*), using CT, found a greater degree of recovery of normal brain size in alcoholic women than in alcoholic men after 2 or more years of abstinence.

## Summary

Alcoholic women experience significant deficits compared with nonalcoholic women over a wide range of neuropsychologic domains. Cognitive skills adversely affected by long-term alcohol abuse include perceptual-motor skills, visual-spatial processes, learning/memory, and abstraction/problem-solving. Alcoholic men and women are impaired to a similar degree, although women typically report shorter or less severe drinking histories. These results suggest that women may be more sensitive to alcohol-related cognitive damage; additional research in this area is essential.

Data are contradictory with respect to alcohol-related changes in brain structure and electrical activity in alcoholic women. Research is needed to explain these inconsistencies and to clarify the relation between neuropsychologic and neurophysiologic functions in alcoholic men and women.

## Footnotes

1 Throughout this article, terms such as “alcohol abuse,” “alcoholism,” and “heavy drinking” are used. There is overlap among these terms in the alcoholism literature; therefore, the wording in each case is based on the terms used in the reference cited.

2 One of the accepted basic characteristics of a scientific model is that it provide a basis for further inquiry.

3 The additional tests included a reaction-time task, a short-term memory test, and a verbal problem-solving task.
